# Supplementary material for: Cross-serotype protection against group A Streptococcal infections induced by immunization with SPy_2191
Source: Nat Commun. 2020 Jul 15;11:3545. doi: 10.1038/s41467-020-17299-x (PMC7363907; doi:10.1038/s41467-020-17299-x)
Supplement: Supplementary file 1 — Supplementary Information [file 41467_2020_17299_MOESM1_ESM.pdf]

Supplementary Information

Cross-serotype protection against group A Streptococcal infections induced by immunization with SPy_2191

Sanduja et al 2020

Supplementary Figures

Cloning, protein expression and purification

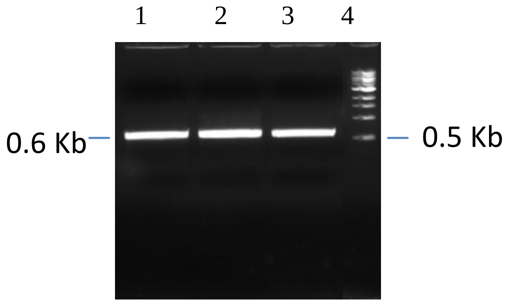

Supplementary Figure 1. PCR amplified gene product were run on 1% agarose gel.
Lane 1 to 3 – PCR amplified SPy_2191 gene & **Lane 4**– 1Kb DNA ladder.

SPy_2191 gene sequence (615 bp)

ATGTTTAAGAAAGAAAATTTAAAACAACGTTATTTTAATTTTGGATTAGTAGCGTTAGCT
CTAACAATATTAGCCATCATTTTTGCCTTCTCAAGTAAAAATGCTGATACTAAGTCTTATG
CTAAGAAGTCAGAAAGTAAAATGGTAACAATCGACAAGGCTCCAAAAAATAATCATGCT
ATTACTAAAGAAGAAAGCAAAGAAAAAGCAAAGAGCATTGCTTCGGAGCCTATTCCCAC
AGTAGAAAACTCTGTAGCTCCGACAGTAACAGAGGAAGCACCGGTTGTTCAGCAAGAAG
TGACTCAAACTGTTCAGCAGGTATCTTCAGTAGCCTATAATCCAAACAATGTGGTACTTT
CCAATGGAAATACTGCTGGTATTGTAGGAAGTCAAGCGGCGGCACAGATGGCAGCAGCA
ACAGGTGTTCCACAATCAACTTGGGAACATATAATTGCGCGTGAATCTAATGGAAATCCT
AACGCAGCTAATGCTTCTGGGGCATCAGGGTTGTTCCAGACAATGCCAGGTTGGGGTTCT
ACAGCAACGGTTGAAGATCAAGTCAATGCAGCCTTGAAAGCCTATAGTGCACAAGGTTT
ATCAGCTTGGGGTTACTAA

Supplementary Figure 2. Sequence of SPy_2191 gene.

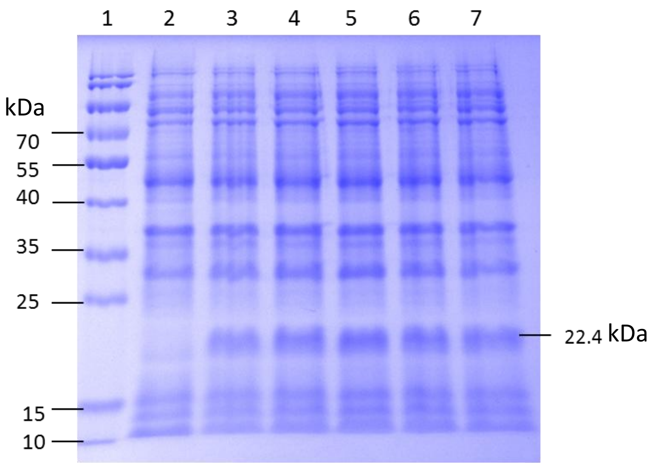

Supplementary Figure 3: SDS-PAGE to check the expression of recombinant protein SPy_2191 in BL21 (DE3) *E. coli* at various concentration of IPTG for 10 hrs induction. **Lane 1** – Protein ladder, **lane 2** – Uninduced expression of SPy_2191. IPTG induced expression of SPy_2191 at different IPTG concentrations, **lane 3** – 0.2 mM, **lane 4** - 0.5 mM, **lane 5** - 1 mM, **lane 6** - 2 mM and **lane 7** - 5 mM.

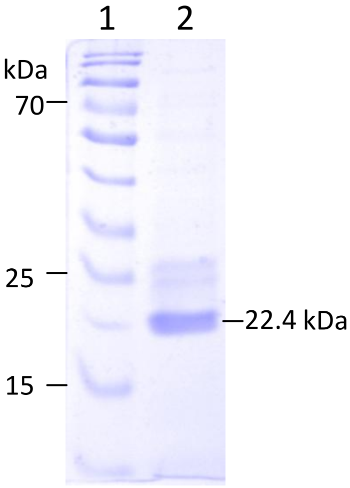

Supplementary Figure 4: SDS-PAGE gel electrophoresis of purified SPy_2191 protein. **Lane 1**, Protein marker **Lane 2**, 22.4 kDa band of SPy_2191 purified protein..

Optimization of vaccine dose:

Before examining its protection in mice, we have determined the optimal concentration of SPy_2191 by subcutaneous route given to C57Bl/6 mice, different amount of SPy_2191 (10µg, 20µg, and 30µg) was injected in mice. We have observed that all the doses were able elicit the same amount of IgG titer (**Supplementary Figure 5**). As we found IgG titer is not dose-dependent, therefore, 10µg was used as vaccine dose for SPy_2191 for immunization.

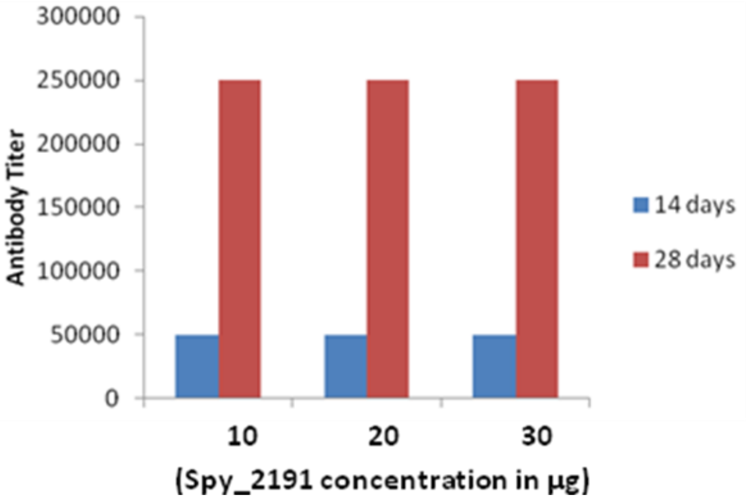

Supplementary Figure 5: IgG titer was determined after subcutaneous immunization with SPy_2191 in C57Bl/6 mice at days 14 and 28.

Supplementary Tables

Supplementary Table 1: List of 45 recombinant antisera generated against selected surface proteins used in this study.

S. No	Sample No.	Sample Name
1.	NP_268607	N-acetylneuraminate-binding proteins
2.	NP_268656	ABC transporter substrate-binding protein
3.	NP_268747	ABC transporter metal binding protein (lipoprotein)
4.	NP_268946	Zinc-binding lipoprotein AdcA precursor
5.	NP_269262	Spermidine/putescine ABC transporter periplasmic transport protein
6.	NP_269379	Phosphate ABC transporter periplasmic phosphate Plasmodium falciparum –binding protein
7.	NP_269403	Putative amino acid ABC transporter, periplasmic amino acid-binding protein
8.	NP_269961	Surface lipoprotein
9.	NP_269968	Laminin adhesion
10.	NP_269421	Maltose/maltodextrin-binding protein
11.	NP_268436	secreted protein/ Hypothetical protein SPy_0019
12.	NP_269203	Extracellular hyaluronate lyase
13.	NP_269467	Internalin A
14.	NP_269488	Foldase PrsA
15.	NP_269743	Esterase
16.	NP_269810	Hypothetical protein SPy_1801/ Immunogenic secreted precursor-like protein
17.	NP_269818	Hypothetical protein SPy_1813
18.	NP_269916	Hypothetical protein SPy_1939
19.	NP_269944	Streptokinase A/ Streptokinase A precursor
20.	NP_269976	Hypothetical protein SPy_2025/ Immunogenic secreted protein precursor
21.	NP_269981	ATP-binding cassette transporter-like protein
22.	NP_269984	Foldase PrsA
23.	NP_270099	Hypothetical protein SPy_2191/ Transglycosylase SLT domain family protein
24.	NP_269045	Hypothetical protein SPy_0836
25.	NP_268639	Penicillin binding protein (D-alanyl-D-alanine carboxypeptidase)
26.	NP_269051	Hypothetical protein SPy_0843
27.	NP_270005	Dipeptidase
28.	NP_270119	Serine protease

29.	NP_268581	Hypothetical protein SPy_0210
30.	NP_269063	Peptidoglycan hydrolase
31.	NP_269570	Hypothetical protein SPy_1492
32.	NP_269625	Hypothetical protein SPy_1558
33.	NP_269940	Pullulanase
34.	NP_268582	Exotoxin G
35.	NP_269402	cAMP factor
36.	NP_268943	Pyrogenic exotoxin C
37.	NP_268860	Hypothetical protein SPy_0604
38.	NP_269208	Hypothetical protein SPy_1037
39.	NP_269417	Hypothetical protein SPy_1290
40.	NP_269569	Hypothetical protein SPy_1491
41.	NP_268542	ABC transporter lipoprotein
42.	NP_269268	Acid phosphatase/ phosphotransferase
43.	NP_268750	Cyclophilin-type protein
44.	NP_268760	Hypothetical protein SPy_0469/ putative 42 KDa protein
45.	NP_269989	Mitogenic factor

Note: All 45 recombinant sera were kind gift of GSK vaccine, Siena, Italy.

Determination of LD50 for different GAS serotype in C57Bl/6 mice for survival assay:

For survival assay study of a selected antigen in mice to check the protection by SPy_2191, we have determined the lethal dose of different serotype of GAS. For LD50 determination, C57Bl/6 mice (n=4) were injected intraperitoneally 100µl bacterial suspension (in PBS) from 10^7, 5×10^7 and 10^8 CFU. Mice survival was recorded daily for 10 days and then they were euthanized. LD50 represents number of days elapsed when percent survival is 50%. The LD50 values of each serotype at a given CFU dose in mentioned. Values in red were used for challenge studies (**Supplementary Table 2**).

Supplementary Table 2. Lethal dose (LD50) calculation of different GAS serotypes CFU in C57Bl/6 mice. The LD50 values of each serotype at a given CFU dose in mentioned.

**GAS serotypes**	**Dose (CFU/mice)**	**LD50 (days)**
M1/IND	10^7	8
	**5×10^7**	3
	10^8	2
M49/IND	10^7	-
	5×10^7	9
	**10^8**	3
M3.1/IL	10^7	-
	5×10^7	5
	**10^8**	2
M1/USA	10^7	12
	5×10^7	6
	**10^8**	3
M1/UK	10^7	3
	**5×10^7**	2
	10^8	1

Values in bold depict doses used for challenge studies.
