# Supplementary material for: Cross-serotype protection against group A Streptococcal infections induced by immunization with SPy_2191
Source: Nat Commun. 2020 Jul 15;11:3545. doi: 10.1038/s41467-020-17299-x (PMC7363907; doi:10.1038/s41467-020-17299-x)
Supplement: Supplementary file 3 — Reporting Summary [file 41467_2020_17299_MOESM3_ESM.pdf]

nature research

Corresponding author(s): Prof. Atul Johri and Prof. Rakesh Bhatnagar

Last updated by author(s): Jun 13, 2020

Reporting Summary

Nature Research wishes to improve the reproducibility of the work that we publish. This form provides structure for consistency and transparency in reporting. For further information on Nature Research policies, see Authors & Referees and the Editorial Policy Checklist .

Statistics

For all statistical analyses, confirm that the following items are present in the figure legend, table legend, main text, or Methods section.

n/a	Confirmed	
☐	☒	The exact sample size (*n*) for each experimental group/condition, given as a discrete number and unit of measurement
☐	☒	A statement on whether measurements were taken from distinct samples or whether the same sample was measured repeatedly
☐	☒	The statistical test(s) used AND whether they are one- or two-sided<br>*Only common tests should be described solely by name; describe more complex techniques in the Methods section.*
☐	☒	A description of all covariates tested
☐	☒	A description of any assumptions or corrections, such as tests of normality and adjustment for multiple comparisons
☐	☒	A full description of the statistical parameters including central tendency (e.g. means) or other basic estimates (e.g. regression coefficient) AND variation (e.g. standard deviation) or associated estimates of uncertainty (e.g. confidence intervals)
☐	☒	For null hypothesis testing, the test statistic (e.g. *F*, *t*, *r*) with confidence intervals, effect sizes, degrees of freedom and *P* value noted<br>*Give P values as exact values whenever suitable.*
☒	☐	For Bayesian analysis, information on the choice of priors and Markov chain Monte Carlo settings
☐	☒	For hierarchical and complex designs, identification of the appropriate level for tests and full reporting of outcomes
☐	☒	Estimates of effect sizes (e.g. Cohen's *d*, Pearson's *r*), indicating how they were calculated

Our web collection on statistics for biologists contains articles on many of the points above.

Software and code

Policy information about availability of computer code

Data collection	No software was used.
Data analysis	GraphPad Prision 6 software, BD CellQuest™ Pro software (Becton Dickinson), FCAP Array™ software, V3.0 (Becton Dickinson).

For manuscripts utilizing custom algorithms or software that are central to the research but not yet described in published literature, software must be made available to editors/reviewers. We strongly encourage code deposition in a community repository (e.g. GitHub). See the Nature Research guidelines for submitting code & software for further information.

Data

Policy information about availability of data

All manuscripts must include a data availability statement. This statement should provide the following information, where applicable:
- Accession codes, unique identifiers, or web links for publicly available datasets
- A list of figures that have associated raw data
- A description of any restrictions on data availability

The data that support the findings of this study are available from the corresponding author upon request.

Field-specific reporting

Please select the one below that is the best fit for your research. If you are not sure, read the appropriate sections before making your selection.

☒ Life sciences ☐ Behavioural & social sciences ☐ Ecological, evolutionary & environmental sciences

For a reference copy of the document with all sections, see nature.com/documents/nr-reporting-summary-flat.pdf

Life sciences study design

All studies must disclose on these points even when the disclosure is negative.

Sample size	Wherever required we have mentioned the sample size in the figure legend of main manuscript and in the supplementary data.
Data exclusions	No data were excluded from the analyses.
Replication	All attempts at replication of data were successful. All the experiments were replicated and performed independently at least twice.
Randomization	For all the experiments, the samples/organisms were allocated to experimental groups or selected at random, to avoid biasness. All the mice were allocated randomly to different groups during all assays.
Blinding	All the investigators were blinded to group allocation during data collection and analysis.

Reporting for specific materials, systems and methods

We require information from authors about some types of materials, experimental systems and methods used in many studies. Here, indicate whether each material, system or method listed is relevant to your study. If you are not sure if a list item applies to your research, read the appropriate section before selecting a response.

Materials & experimental systems

n/a	Involved in the study	
☐	☒	Antibodies
☐	☒	Eukaryotic cell lines
☒	☐	Palaeontology
☐	☒	Animals and other organisms
☒	☐	Human research participants
☒	☐	Clinical data

Methods

n/a	Involved in the study	
☒	☐	ChIP-seq
☐	☒	Flow cytometry
☒	☐	MRI-based neuroimaging

Antibodies

Antibodies used	Goat anti-mouse IgG conjugated with FITC (Thermo Scientific, A16067), Goat anti-mouse IgG HRP (Abcam, ab6789). The polyclonal antibodies used in this study were not commercially purchased. They were rather generated for research purpose by GSK Vaccine and obtained as a gift for the present study.
Validation	Based on the indirect ELISA, derives its specificity and sensitivity by employing high affinity capture and detection antibodies and enzyme-amplification. The primary antibodies used in this study were either raised in mice in the present study or obtained as a gift from GSK vaccine.

Eukaryotic cell lines

Policy information about cell lines

Cell line source(s)	HEp2 cell line from National Centre for Cell Science, University of Pune, India
Authentication	Short Tandem Repeat (STR) analysis was used for the authentication.
Mycoplasma contamination	Cell line was tested negative for mycoplasma contamination.
Commonly misidentified lines (See ICLAC register)	No misidentified cell line was procured or used in the present study.

Animals and other organisms

Policy information about studies involving animals; ARRIVE guidelines recommended for reporting animal research

Laboratory animals	Six weeks old, specific-pathogen-free, inbred, female C57BL/6 mice were purchased from Hylasco Biotechnology India Pvt. Ltd. Mice were housed within a Biosafety level-3 unit, within ventilated micro-isolator cages, fed pelleted diet and water ad libitum. Housing conditions- 23'C temperature, 50% humidity, with 12-hour light/12-hour dark cycle.
Wild animals	The study did not involve wild animals.
Field-collected samples	Study did not involve samples collected form the field.

Ethics oversight	Mice infection studies were carried out in strict accordance with the recommendations given by the Institutional Animal Ethics Committee (IAEC), Jawaharlal Nehru University and Council for the Purpose of Control and Supervision of Experiments on Animals (CPCSEA, Ministry of Social Justice and Empowerment, Government of India), New Delhi. Protocols were approved by IERB board and all efforts were made to minimize the suffering of mice employed in the study with IAEC code 10/2017.

Note that full information on the approval of the study protocol must also be provided in the manuscript.

Flow Cytometry

Plots

Confirm that:

- [x] The axis labels state the marker and fluorochrome used (e.g. CD4-FITC).
- [x] The axis scales are clearly visible. Include numbers along axes only for bottom left plot of group (a 'group' is an analysis of identical markers).
- [x] All plots are contour plots with outliers or pseudocolor plots.
- [x] A numerical value for number of cells or percentage (with statistics) is provided.

Methodology

Sample preparation	Study 1: For surface localization, Group A streptococcus bacterial cells were used in flow cytometry was grown in THY (Todd-Hewit broth media with yeast extract) till OD600 reaches 0.4.<br>Study 2: For cytokine level measurement, ex-vivo cultured splenocytes was assessed by measuring the level of different cytokines released in the culture supernatant after antigen re-stimulation. Two weeks after the final booster (day 42), mice were euthanized; splenocytes were isolated and stimulated with SPy_2191 antigen for 72 h. The BD™ Cytometric Bead Array (CBA) mouse Th1/Th2/Th17 cytokine kit (BD Biosciences) was used for estimation of different cytokine in the culture supernatant.
Instrument	For study 1: FACS Calibur cytometer (Becton Dickinson) and for study 2: BD FACSCanto-II flow cytometer
Software	For study 1: Cell quest software was used and for study 2: FCAP Array software, V3.0
Cell population abundance	Pure sample of group A streptococcus was used and is determined by Remel Streptex and acid extraction kit.
Gating strategy	Study 1: For each candidate, we have recorded mean fluorescence intensity with both immune sera and pre-immune sera. Mean fluorescence intensity ratio of immune sera/ pre immune sera forms the x-axis (fold difference in mean fluorescence intensity). We predicted that antigen showing > 2.4 fold difference in mean fluorescence intensity would be sufficient to be surface exposed on a bacterial surface. Therefore the red dotted gate is defined as the minimum fold intensity required by an antigen to be surface exposed.<br><br>Study 2: Bead based method, with internal controls that required to make standard curve for quantification of cytokines. No gating required.

- [ ] Tick this box to confirm that a figure exemplifying the gating strategy is provided in the Supplementary Information.
